# Strain parameters for predicting the prognosis of non‐ischemic dilated cardiomyopathy using cardiovascular magnetic resonance tissue feature tracking

**DOI:** 10.1186/s12968-021-00726-3

**Published:** 2021-03-15

**Authors:** Chengjie Gao, Yajie Gao, Jingyu Hang, Meng Wei, Jingbo Li, Qing Wan, Yijing Tao, Hao Wu, Zhili Xia, Chengxing Shen, Jingwei Pan

**Affiliations:** Department of Cardiology, Shanghai Jiao Tong University Affiliated Sixth People’s Hospital, No.600 Yishan Road, Xuhui District, Shanghai, China

**Keywords:** Heart failure, Non‐ischemic dilated cardiomyopathy, Cardiovascular magnetic resonance, Prognosis

## Abstract

**Background:**

A considerable number of non-ischemic dilated cardiomyopathy (NDCM) patients had been found to have normalized left ventricular (LV) size and systolic function with tailored medical treatments. Accordingly, we aimed to evaluate if strain parameters assessed by cardiovascular magnetic resonance (CMR) feature tracking (FT) analysis could predict the NDCM recovery.

**Methods:**

79 newly diagnosed NDCM patients who underwent baseline and follow-up CMR scans were enrolled. Recovery was defined as a current normalized LV size and systolic function evaluated by CMR.

**Results:**

Among 79 patients, 21 (27%) were confirmed recovered at a median follow-up of 36 months. Recovered patients presented with faster heart rates (HR) and larger body surface area (BSA) at baseline (P < 0.05). Compared to unrecovered patients, recovered pateints had a higher LV apical radial strain divided by basal radial strain (RS_api/bas_) and a lower standard deviation of time to peak radial strain in 16 segments of the LV (SD16-TTPRS). According to a multivariate logistic regression model, RS_api/bas_ (P = 0.035) and SD16-TTPRS (P = 0.012) resulted as significant predictors for differentiation of recovered from unrecovered patients. The sensitivity and specificity of RS_api/bas_ and SD16-TTPRS for predicting recovered conditions were 76%, 67%, and 91%, 59%, with the area under the curve of 0.75 and 0.76, respectively. Further, Kaplan Meier survival analysis showed that patients with RS_api/bas_ ≥ 0.95% and SD16-FTPRS ≤ 111 ms had the highest recovery rate (65%, P = 0.027).

**Conclusions:**

RS_api/bas_ and CMR SD16-TTPRS may be used as non-invasive parameters for predicting LV recovery in NDCM. This finding may be beneficial for subsequent treatments and prognosis of NDCM patients. *Registration number*: ChiCTR-POC-17012586.

## Background

Non-ischemic dilated cardiomyopathy (NDCM) is a condition that manifests as the enlargement of the left ventricle (LV) or both ventricles with systolic dysfunction or abnormal loading conditions [1]. As one of the leading causes of systolic heart failure (HF), NDCM principally affects young adults, leading to tragic outcomes. Early diagnosis, standard therapy, and timely follow-up have led to remarkable achievements in NDCM patients’ prognosis, with an increase of 5-year free event survival rate from 62 to 93% over the past three decades [2]. Several studies have recently reported that numbers of NDCM patients, including children and adults, have experienced drastic improvement of LV systolic function with tailored medical treatments on serial echocardiography follow-up [3–6].

With excellent spatial resolution and high reproducibility, cardiovascular magnetic resonance (CMR) is considered the non-invasive gold standard for evaluating cardiac function and myocardial contraction [7]. Nowadays, CMR can provide a more specific diagnosis of cardiomyopathies than conventional techniques [8]. Precise assessment and diagnosis could have important clinical implications, especially in preventing clinical adverse events, choosing optimal treatment regimens, and timing of transplantation in cardiomyopathy patients. Myocardial strain analysis or feature tracking (FT) imaging provides more sophisticated information on cardiac function over and beyond conventional CMR derived volumes and global contractile function (LV ejection fraction [LVEF]) [9]. Most studies investigating regional LV function and motion abnormalities have focused on longitudinal dimension [10, 11]. Yet, the largest degree of myocardial deformation occurs in the radial direction, so that radial deformation and radial synchrony are more likely to be sensitive markers for predicting the prognosis of NDCM patients. Few studies have reported deformation assessment as a prognosticator in reversible NDCM patients. The purpose of this study was to evaluate if strain parameters assessed by CMR FT analysis could predict the reversible NDCM.

## Methods

### Study population

A total of 120 newly diagnosed (symptoms occurred within 2 months) NDCM patients based on the 1995 World Health Organization/International Society and Federation of Cardiology criteria [12] in our heart center from January 1st 2011 to June 30th 2016 were consecutively enrolled. Inclusion criteria were impaired systolic function (CMR LV ejection fraction (LVEF) ≤ 45%). Exclusion criteria were: (1) ≥ 50% stenosis of a major coronary artery or branch based on invasive coronary angiography or computed tomography angiogram; (2) estimated glomerular filtration rate (eGFR) < 30 ml/min/1.73 m^2^; (3) HF secondary to chronic lung disease; (4) valvular disease of moderate or greater severity; (5) active myocarditis; (6) other cardiomyopathies (ischemic; stress-related; tachycardia; peripartum; metabolic or endocrine diseases) [13]. In addition, patients with any contraindications failed to accomplish CMR test (n = 3), CMR images of inadequate quality (n = 3), implantation of cardiac resynchronization and/or defibrillator therapies or LV assist devices unable to accomplish the follow-up CMR test (n = 31) and heart transplantation (n = 4) were excluded. After exclusion, 79 patients were included in the final analysis. At enrollment, detailed medical data were obtained from all patients, including physical examination, blood laboratory tests, 12-lead electrocardiogram (ECG), and echocardiography. Optimal medical treatments were prescribed and maintained over follow-up.

A median interval of 36 months (interquartile range [IQR], 30 to 48 months) between the enrollment and follow-up CMR(n = 79) were used to classify patients into two groups. Recovery was defined as a current LVEF of ≥ 50% and a CMR LV end-diastolic volume indexed to body surface area (LVEDVI) within the normal range; and plasma N-terminal pro-B-type natriuretic peptide (NT-proBNP) concentration less than 250 ng/L [13, 14]. The remaining NDCM patients were classified as unrecovered. Another 25 healthy healthy subjects without any other primary diseases and abnormalities of CMR images were selected from our CMR imaging database.

The study was approved by the Ethics Committee of Shanghai Jiaotong University Affiliated No.6 Hospital. Written informed consent was obtained from all participants.

### CMR technique

ECG-gated CMR studies were performed on a 3T CMR system (Philips Healthcare, Best, The Netherlands). Balanced steady-state free precision (bSSFP) cines were acquired in three long-axis slices (each plane with 60 degrees interval along the central axis of the LV; four-, three- and two-chamber). Subsequent short-axis cines extending from the mitral valve ring to the apex were obtained to cover the entire LV (8 mm parallel slices with no gap; TR = 3.2 ms, TE = 1.5 ms, flip angle 45, an in-plane resolution was 1.9 × 1.9 mm^2^, acquisition matrix 232 × 219; 30 phases per cardiac cycle). Late gadolinium enhancement (LGE) imaging was performed 10 min after injection of 0.2 mmol/kg of contrast (gadobutrol/Gadovist; Bayer Healthcare, Berlin, Germany).

### CMR analysis

The analysis of LV volumes (end-diastolic volume (LVEDV); end-systolic volume (LVESV)), LVEF, right ventricular (RV) ejection fraction (RVEF), and LV mass (LVM) were performed using standardized protocols and dedicated software (cvi42, Circle Cardiovascular Imaging, Calgary, Alberta, Canada). LVM was estimated at end-diastole and corroborated at end-systole, which excluded the papillary muscles. LV cardiac index (CI) was calculated according to the formula below: CI = $\frac{LVEDV-LVESV}{BSA\times1000}\times HR$. Height and weight were measured in all patients, and body surface area (BSA) was calculated using the Mosteller formula [15].

CMR feature tracking (FT) analysis was performed on the standard acquired bSSFP cine images using cvi42 (Circle Cardiovascular Imaging). LV endocardial and epicardial borders were automatically tracked and manually corrected in three long-axis slices and in short-axis slices in the end-diastolic phase. RV endocardial and epicardial borders were manually traced in the end-diastolic phase in long-axis slices and in short-axis slices. Left atrial (LA) endocardial and epicardial borders were manually traced in the apical four-chamber and apical two-chamber views at end-systole [16]. Global LV and RV global longitudinal strain (GLS) was derived from the long-axis cines, while short-axis cines were used to deduce global LV and RV global circumferential (GCS) and global radial strains (GRS) [17]. Segmental LV peak circumferential strain (CS) and radial strain (RS) were obtained from three consecutive parts (basal, middle, apical) from the mitral annulus to apex. To reflect the base-to-apex CS and RS gradient, the ratios of apical, basal CS and RS (CS_api/bas_, RS_api/bas_) were calculated as apical strain divided by basal strain, respectively. Instantaneous LV peak torsion was defined as the maximum difference in rotation angle between the base and apex divided by the distance between apical and basal slices automatically (Additional file 1). Dyssynchrony was assessed by variability in time to peak strain. The SD16-TTPLS, SD16-FTPCS, and SD16-FTPRS were calculated based on the standard deviation of time to peak longitudinal strain (LS), CS, and RS in 6 basal, 6 mid-ventricle, and 4 apical segments of the LV, respectively (16 segments in all). LA strain values for each tissue point and the reservoir strain values were automatically derived.

Quantitative assessment of myocardial fibrosis was performed on LGE imaging data on short-axis images using cvi42 (Circle Cardiovascular Imaging). Normal myocardium was visually defined as a region of myocardium without any apparent LGE on visual inspection. The mean signal intensity and standard deviation (SD) were determined by drawing a region of interest (ROI) in a portion of the normal myocardium on each slice. The semi-automated greyscale threshold technique was performed by using 2, 4, 6 SD above the mean signal intensity for the normal nulled myocardium. Results were reported as the percentage of LGE mass to the total LVM [18].

### Intra- and inter-observer agreement

Data from 10 healthy subjects and 10 NDCM patients were applied to test inter- and intraobserver variability. 2 independent cardiologists (***1 and ***2) specialized in CMR were blinded to each other’s recordings and conducted separate CMR analyses. All CMR measurements were analyzed by both observers. Data from the separate acquisitions were used to test interobserver variability. In order to test intraobserver variability, the observers re-analyzed their own recordings (3 weeks apart).

### Statistical analysis

Summary statistics of clinical and image data were expressed as mean ± SD, median (quartile25–quartile75) or percentage, as appropriate. Comparisons between groups were made using one-way ANOVA analysis for continuous, normally distributed data and Wilcoxon rank-sum test for continuous, non-normally distributed data. The bonferroni correction was made for multiple comparisons. The chi-square test or Fisher’s exact test was calculated for categorical variables. Univariate and multivariate logistic regression analyses were performed to identify prognostic predictors for the recovered condition. Considering the collinearity and clinical significance, 4 variables (heart rate (HR), BSA, RS_api/bas_, SD16-FTPRS) were included in the model with the predictive accuracy of 81%. Receiver-operating characteristic curve analysis was used to identify parameters that were best fit in diagnosing the recovered model of NDCM. The best cutoff value was based on the maximum Youden index. The Kaplan Meier survival analysis was applied to calculate the recovery rate. The intraclass correlation coefficient (ICC) was used to determine inter- and intraobserver reproducibility. All calculations were performed using SPSS (version 22.0, Statistical Package for the Social Sciences, International Business Machines, Inc., Armonk, New York, USA and GraphPad Prism (version 8.0, Graph-Pad Software, San Diego, California, USA).

## Results

### Baseline clinical characterization of recovered NDCM

**Fig. 1:**
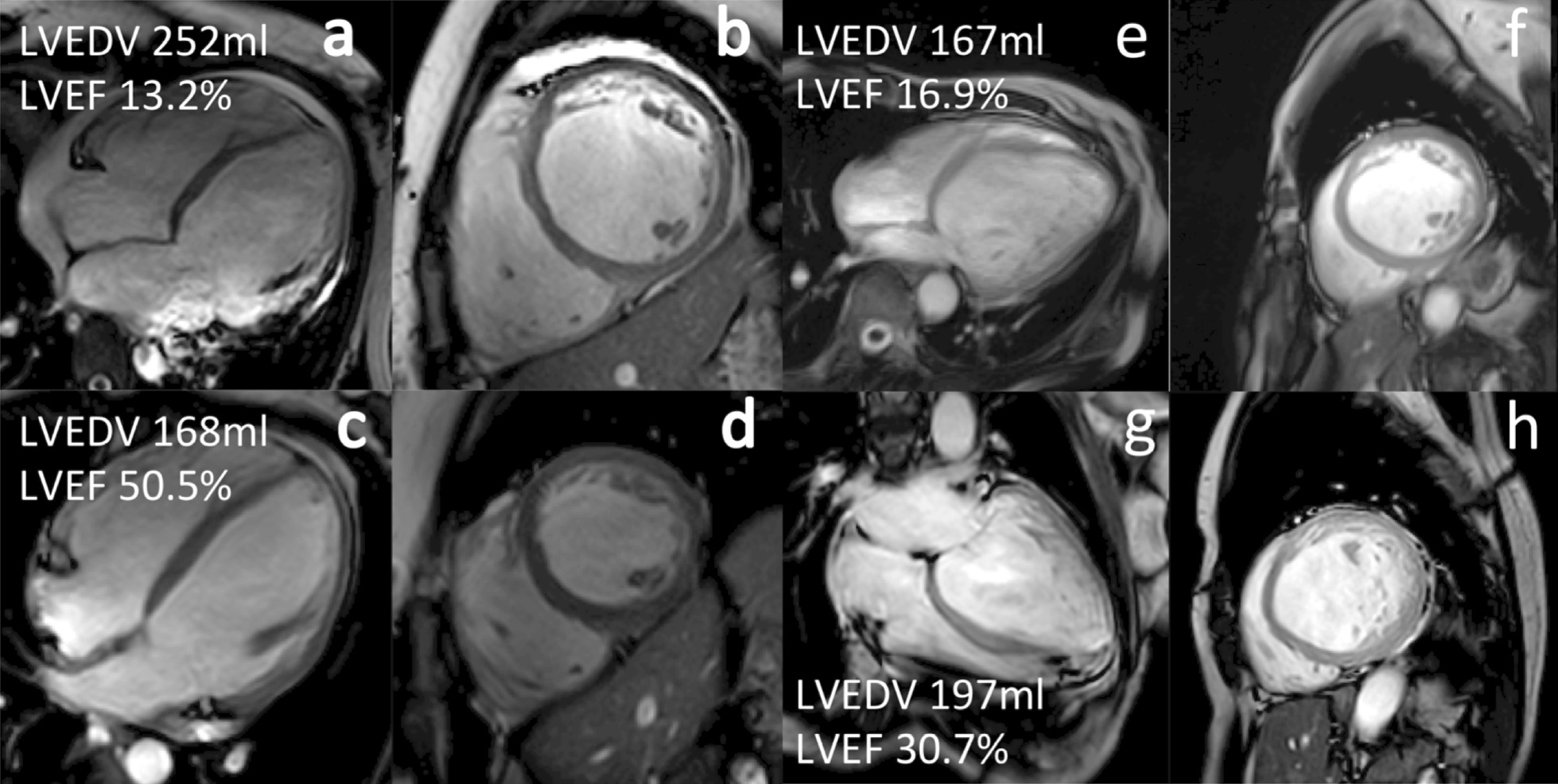
Four chamber cine view and short axis cine view of recovered NDCM (**a**–**d**) and unrecovered NDCM (**e**–**h**)

Baseline CMR test, available baseline, and follow-up data from 79 NDCM patients were analyzed (51 ± 16 years; 77% men). Recovery in both LV size and LVEF was observed in 21 out of 79 patients (46 ± 14 years; 90% men) compared with the other 58 patients (53 ± 16 years; 72% men). No differences in medications were observed between the recovered and unrecovered group. The recovered group patients showed faster baseline HR (81 ± 19 versus 70 ± 17; p = 0.016), larger BSA (1.99 ± 0.30 versus 1.85 ± 0.24; p = 0.027), and higher hemoglobin (Hb) (148 ± 17 versus 140 ± 16; p = 0.039). The baseline clinical variables of the study groups are presented in Table 1. The recovered and unrecovered patients four-chamber and short-axis cine images are shown in Fig. 1.

**Table 1:** Baseline characteristics of the study population

	Healthy control subjects (n = 25)	Recovered NDCM (n = 21)	Unrecovered NDCM (n = 58)	P value
Age, y	48 ± 11	46 ± 14	53 ± 16	0.064
Male, %	18 (72%)	19 (90%)	42 (72%)	0.219
Systolic BP, mmHg	120 ± 11	124 ± 19	122 ± 16	0.603
Diastolic BP, mmHg	74 ± 9	81 ± 16	77 ± 12	0.178
Heart rate, beats/min	74 ± 11	81 ± 19^§^	70 ± 17	0.016
Body surface area, m^2^^2^	1.80 ± 0.25	1.99 ± 0.30^†§^	1.85 ± 0.24	0.027
NYHA classes				
I–II%	−	9 (43%)	29 (50%)	0.575
III–IV%	−	12 (57%)	29 (50%)	
Laboratory characteristics				
Hemoglobin, g/L	146 ± 9	148 ± 17^§^	140 ± 16	0.039
Scr, mmol/L	78 ± 16	85 ± 19	83 ± 17	0.775
Baseline NT-proBNP, ng/l	69 (49,78)	1818 (817,5151)^†^	1598 (739,3662)^†^	0.450
Follow-up NT-proBNP, ng/l	−	69 (22,131)^§^	880 (286,1905)	< 0.001
Electrocardiogram variables				
QRS duration, ms	87 ± 7	91 ± 15	99 ± 23^†^	0.103
Baseline CMR parameters				
LVMI, g/m^2^	54 ± 10	81 ± 21^†^	87 ± 28^†^	0.293
LVEDVI, ml/m^2^^2^	68 ± 11	129 ± 29^†^	140 ± 44^†^	0.228
LVESVI, ml/m^2^	29 ± 6	94 ± 26^†^	106 ± 36^†^	0.119
LVSVI, ml/m^2^	40 ± 12	35 ± 9	34 ± 13^†^	0.695
LVEF, %	58 ± 5	28 ± 7^†^	25 ± 7^†^	0.080
LVCI, L/min/m^2^	2.94 ± 0.74	2.80 ± 0.87	2.45 ± 0.93^†^	0.131
RVEF, %	54 ± 6	41 ± 6^†^	42 ± 9^†^	0.613
LGE, +% (visual)	−	5 (24%)	24 (41%)	0.152
LGE quantitative, 2 SD%	−	24.8 ± 13.0	28.1 ± 16.7	0.415
LGE quantitative, 4 SD%	−	8.8 (4.2,17.3)	12.3 (2.9,21.1)	0.842
LGE quantitative, 6 SD%	−	3.5 (1.2,9.0)	5.2 (0.7,11.4)	0.868
Follow-up CMR parameters				
LVMI, g/m^2^	−	66 ± 15^§^	84 ± 31	0.014
LVEDVI, ml/m^2^	−	79 ± 18^§^	118 ± 37	< 0.001
LVESVI, ml/m^2^	−	34 ± 10^§^	81 ± 32	< 0.001
LVSVI, ml/m^2^	−	45 ± 9	38 ± 20	0.123
LVEF, %	−	57 ± 5^§^	31 ± 10	< 0.001
LVCI, L/min/m^2^	−	3.26 ± 0.60^§^	2.64 ± 1.35	0.047
RVEF, %	−	48 ± 5	46 ± 9	0.094
Medications				
ACEI/ARB, %	−	20 (95%)	53 (91%)	0.567
Beta-blocker, %	−	21 (100%)	57 (98%)	0.545
Spironolactone, %	−	18 (86%)	52 (90%)	0.626
Diuretics, %	−	18 (86%)	52 (90%)	0.626
Digoxin, %	−	4 (19%)	6 (12%)	0.325
Comorbidities				
Diabetes mellitus, %	−	5 (24%)	13 (22%)	0.896
Hypertension, %	−	11 (52%)	20 (34%)	0.304

*BP* blood pressure, *NYHA* New York Heart Association, *Scr* serum creatinine, *NT-proBNP* plasma N-terminal pro-B-type natriuretic peptide, *LVMI* left ventricular mass indexed to body surface area, *LVEDVI* left ventricular end diastolic volume indexed to body surface area, *LVESVI* left ventricular end systolic volume indexed to body surface area, *LVSVI* left ventricular stroke volume indexed to body surface area, *LVEF* left ventricular ejection fraction, *LVCI* left ventricular cardiac index, *RVEF* right ventricular ejection fraction, *LGE* late gadolinium enhancement, *ACEI* angiotensin-converting enzyme inhibitors, *ARB*, angiotensin receptor blocker

^†^P < 0.05 vs. control

^§^P < 0.05 vs. Unrecovered NDCM, P values for baseline characteristics of the recovered and unrecovered patients were presented

### Baseline and follow‐up LV systolic function evaluation by CMR

**Fig. 2:**
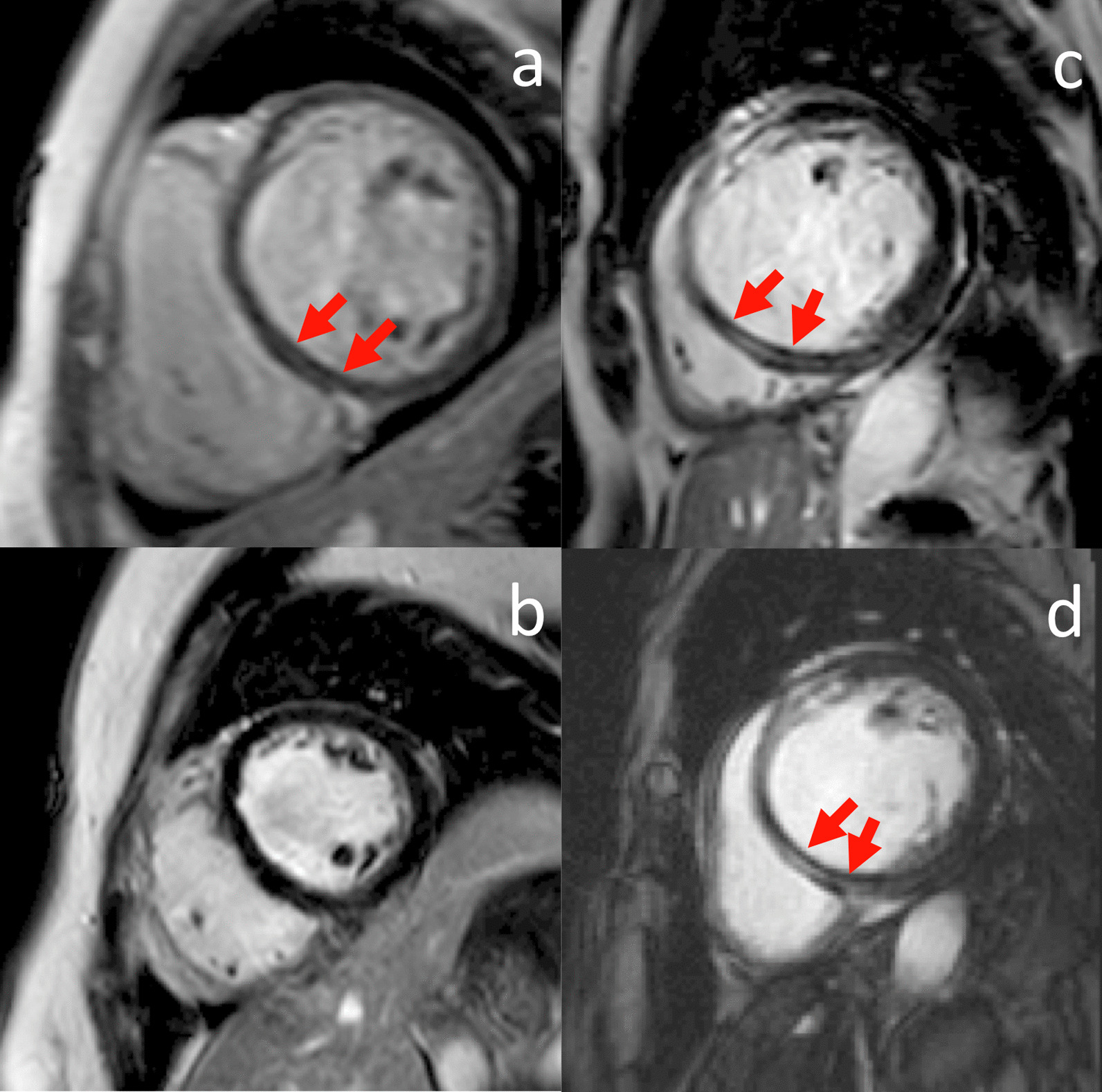
Baseline and follow-up LGE in recovered (**a**, **b**) and unrecovered (**c**, **d**) NDCM patients

**Fig. 3:**
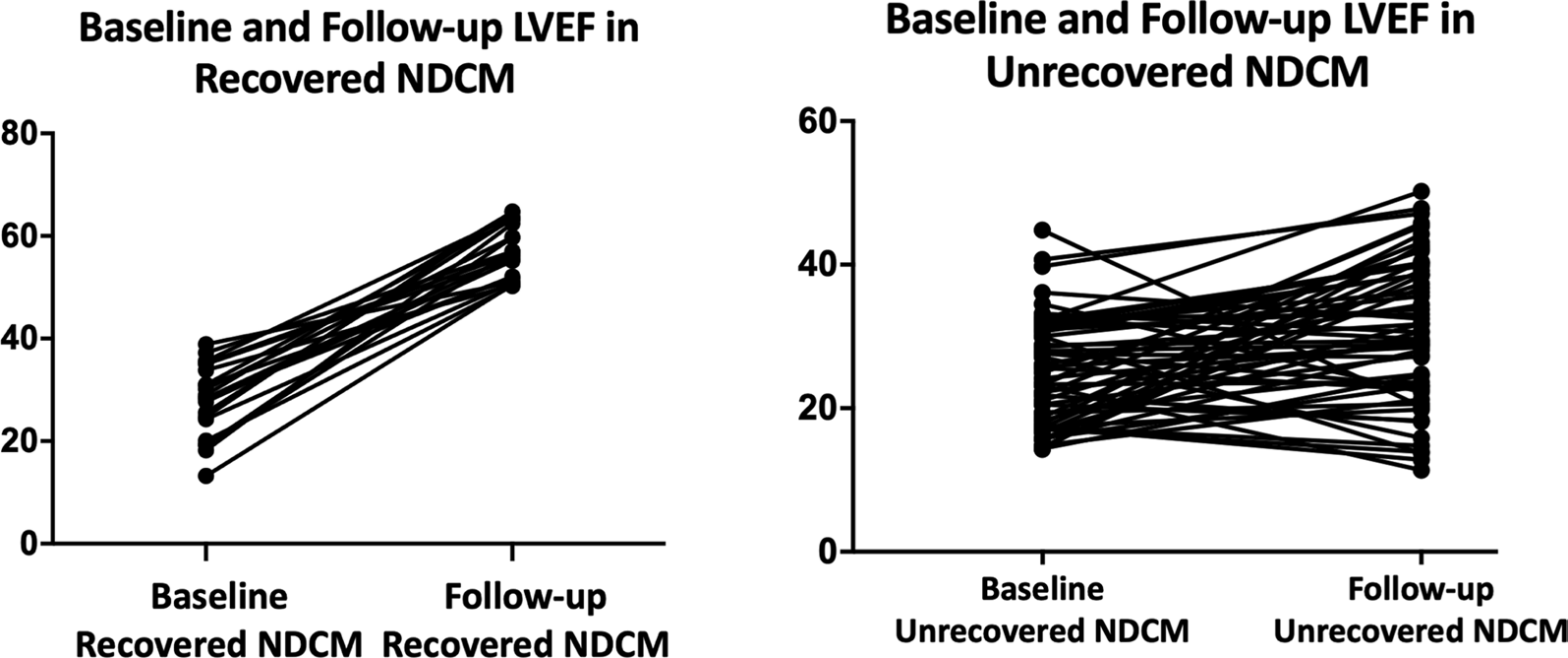
Baseline and follow-up LVEF in recovered and unrecovered NDCM

Baseline CMR parameters including LVEDVI, LVESV indexed to BSA (LVESVI), LVEF, LV stroke volume indexed to BSA (SVI), CI, LVM indexed to BSA (LVMI), and RVEF were similar between two NDCM groups. The qualitative LGE analysis was also similar. While follow-up CMR parameters of the recovered patients, including LVMI, LVEDVI, LVESVI, LVEF, and CI, were closer to those observed in healthy control subjects and were different when compared with unrecovered NDCM patients. CMR parameters are listed in Table 1. Baseline and follow-up LGE of recovered and unrecovered NDCM patients are shown in Fig. 2. Baseline and follow-up LVEF of recovered and unrecovered NDCM patients evaluated by CMR are shown in Fig. 3.

### CMR global and segmental strains

**Fig. 4:**
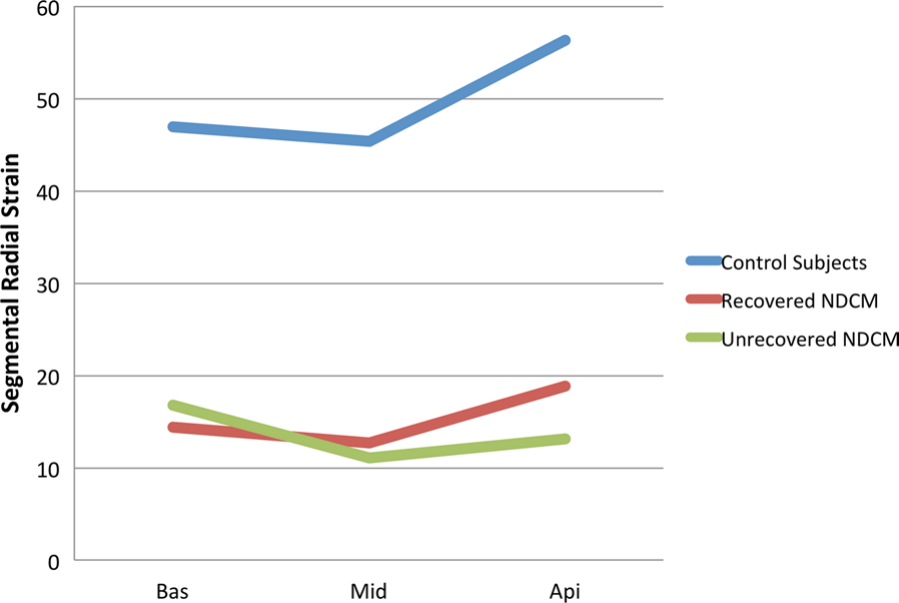
Comparing with control subjects, the absolute value of left ventricular segmental radial strain in NDCM patients was obviously lower, but the base-to-apex radial strain gradient in recovered group was preserved. Unrecovered patients showed more severe injury at apical part

Global LV, RV strains, peak systolic torsion, and LA reservoir strain were severely decreased in both NDCM groups compared with the healthy subjects. LV CS and RS of the basal and middle parts (CS_bas_, CS_mid,_ and RS_bas_, RS_mid_) were similar between two NDCM groups, but the apical strains were significantly lower in the unrecovered group (Fig. 4). As segmental strain was an absolute index with individuality, the self-correction index was used to reflect the relative variation of strain. The CS_api/bas_ and RS_api/bas_ ratios were significantly lower in unrecovered patients compared to recovered ones. RS_api/bas_ showed differences among three groups; RS_api/bas_ was the highest in recovered NDCM, followed by the healthy control group, while it was the lowest in unrecovered NDCM. Strain parameters are shown in Table 2.

**Table 2:** Global and segmental strain assessed by cardiovascular magnetic resonance

	Healthy control subjects (n = 25)	Recovered NDCM (n = 21)	Unrecovered NDCM (n = 58)	P value
LV-GLS, %	− 20.2 ± 2.2	− 8.4 ± 3.5^†^	− 7.6 ± 3.2^†^	0.313
SD16-TTPLS, ms	84 ± 32	96 ± 30	126 ± 53^†^	0.008
LV-GCS, %	− 21.9 ± 2.9	− 8.5 ± 4.0^†^	− 7.4 ± 3.0^†^	0.190
bas, %	− 21.7 ± 3.1	− 8.7 ± 3.0^†^	− 9.4 ± 3.6^†^	0.364
mid, %	− 21.8 ± 3.6	− 8.3 ± 4.4^†^	− 7.1 ± 3.3^†^	0.216
api, %	− 24.0 ± 3.1	-11.2 ± 5.1^†^	− 8.5 ± 4.4^†^	0.017
CS_api/bas_	1.12 ± 0.18	1.40 ± 0.74^§^	0.97 ± 0.47	0.001
SD16-TTPCS, ms	67 ± 14	87 ± 28^§^	125 ± 60^†^	0.002
LV-GRS, %	47.9 ± 9.4	14.5 ± 7.5^†^	12.3 ± 6.5^†^	0.262
bas, %	47.0 ± 13.5	14.4 ± 6.0^†^	16.8 ± 9.6^†^	0.347
mid, %	45.3 ± 12.6	12.7 ± 7.3^†^	11.0 ± 6.2^†^	0.440
api, %	56.4 ± 11.5	18.8 ± 10.2^†^	13.2 ± 8.2^†^	0.021
RS_api/bas_	1.22 ± 0.37	1.44 ± 0.76^§^	0.92 ± 0.59	0.001
SD16-TTPRS, ms	65 ± 19	85 ± 20^§^	129 ± 66^†^	0.001
Peak systolic torsion, deg/cm	2.85 ± 1.83	2.01 ± 1.09^†^	1.96 ± 1.06^†^	0.858
RV-GLS, %	− 23.6 ± 5.5	− 15.9 ± 5.2^†^	− 14.7 ± 7.3^†^	0.475
RV-GCS, %	− 12.1 ± 2.6	− 6.7 ± 3.4^†^	− 5.7 ± 4.4^†^	0.303
RV-GRS, %	23.5 ± 8.2	11.7 ± 5.7^†^	12.2 ± 6.5^†^	0.782
LA-reservoir, %	36.1 ± 12.6	15.4 ± 6.9^†^	15.5 ± 10.8^†^	0.988

*LV* left ventricular, *GLS* global longitudinal strain, *SD16-TTPLS* standard deviation of time to peak longitudinal strain in LV 16 segments, *GCS* global circumferential strain, *CS*_*api/bas*_ apical circumferential stain divided by basal circumferential strain, *RS*_*api/bas*_ apical radial stain divided by basal radial strain, *SD16-TTPCS* standard deviation of time to peak circumferential strain in LV 16 segments, *GRS* global radial strain, *SD16-TTPRS* standard deviation of time to peak radial strain in LV 16 segments, *RV* right ventricular, *LA* left atrial

^†^P < 0.003 vs. control

^§^P < 0.003 vs. Unrecovered NDCM (Bonferroni correction), P values for strain parameters assessed by CMR of the recovered and unrecovered patients were presented

### Electric and mechanical synchronism

**Fig. 5:**
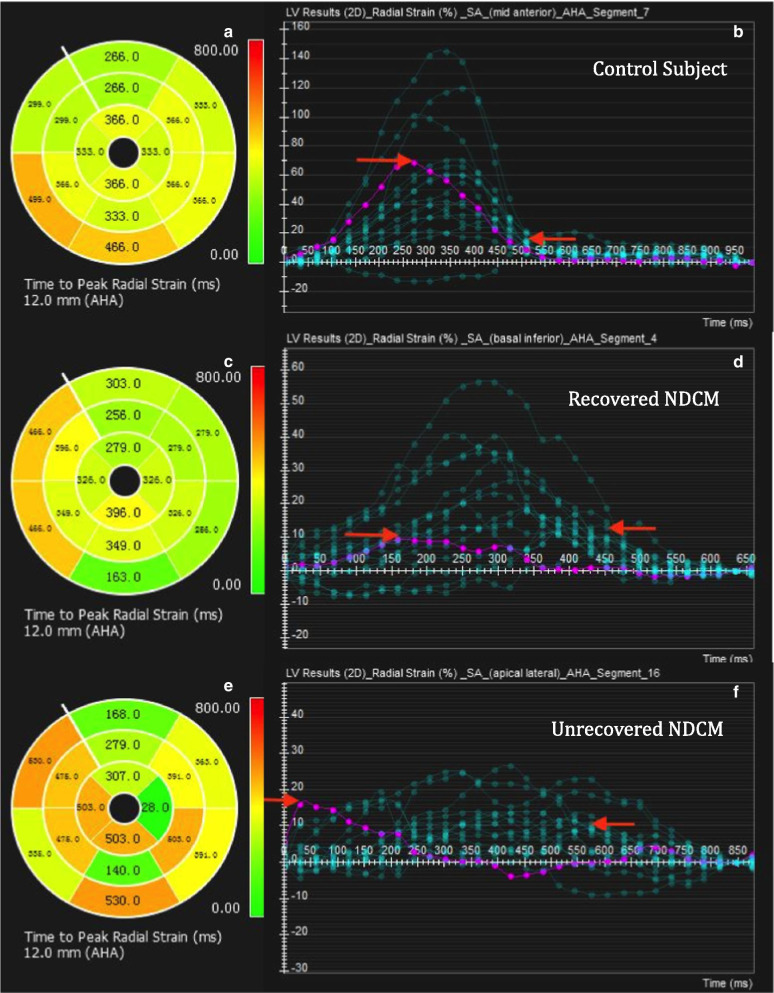
The left panels are bull eyes patterns of time to peak radial strain of left ventricle subdivided into 16 segments in control subject (**a**), recovered NDCM patient (**c**) and unrecovered NDCM patient (**e**). SD16-TTPRS was 60 ms, 76 ms and 158 ms, respectively. Compared with control subjects, the time gap was prolonged in recovered NDCM and was the largest in the unrecovered NDCM cohort. This led ineffective shift of blood and lower left ventricular stroke volume. The right panels demonstrated peak radial stain curves for synchronism, which showed homogeneity in control subject (**b**), less coincidence in recovered NDCM (**d**) and the most inconformity in unrecovered NDCM (**f**). The purple curve in each graph indicated the earliest segment reaching the peak radial strain, which was segment 7, 4 and 16, respectively. The left red arrow in each graph was the time the earliest segment reached the peak radial strain, whereas red arrow in the right was the time of the latest segment. The time gap gradually increased in healthy control subjects, recovered NDCM and unrecovered NDCM

QRS duration by ECG reflects electric synchronism between groups, and was similar (Table 1). Meanwhile, the standard deviation of time to peak longitudinal strain in LV 16 segments (SD16-FTPLS), the standard deviation of time to peak circumferential strain in LV 16 segments (SD16-TTPCS), and standard deviation of time to peak radial strain in LV 16 segments (SD16-TTPRS) were calculated to reflect intraventricular mechanical synchronism, which was highly consistent in healthy control subjects (SD16-TTPCS: 68 ± 14 ms; SD16-TTPRS: 65 ± 19 ms, respectively). SD16-TTPCS and SD16-TTPRS in the recovered group were much more coincident compared to the unrecovered group (Table 2) (SD16-FTPCS: 87 ± 28 ms versus 125 ± 60 ms, P = 0.002; SD16-TTPRS: 85 ± 20 ms versus 129 ± 66 ms; P = 0.001). Figure 5 illustrates TT-derived peak radial strain examples in 16 segments from a control subject, a recovered NDCM patient, and an unrecovered NDCM patient.

### Predictor of the recovered condition in NDCM patients

**Fig. 6:**
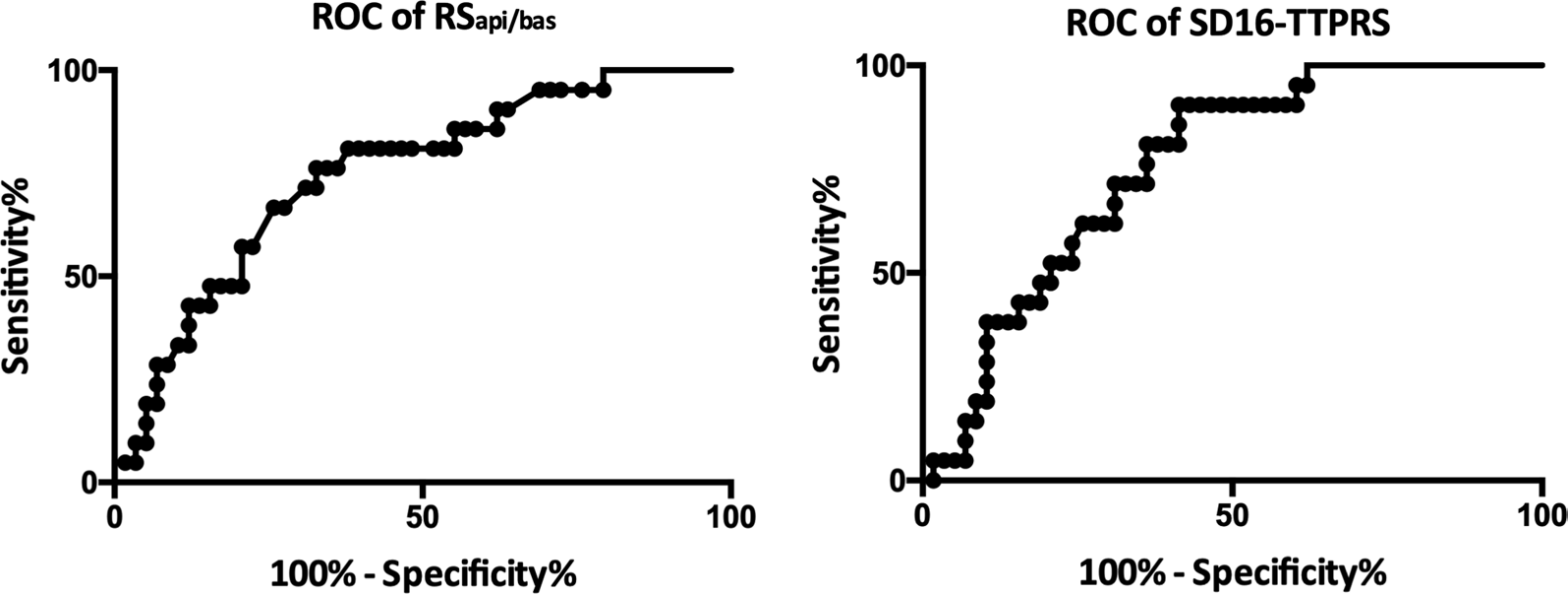
The cutoff point of RS_api/bas_ and SD16-TTPRS for predicting the recovered condition was 0.95 and 111 ms, respectively. The sensitivity and specificity for prediction of left ventricular size and systolic functional recovery were 76%, 67% (95%CI 0.63–0.87) and 91%, 59% (95%CI 0.65–0.87) respectively. The area under curve was 0.75 and 0.76

To determine the predictor of recovered NDCM patients, multivariate analysis was performed of association between baseline clinical data, CMR variables, and recovered condition. Based on univariate logistic regression analysis, the recovered condition was correlated with HR, BSA, RS_api/bas_, SD16-TTPRS, CS_api/bas_ and SD16-TTPPCS. Considering the collinearity and clinical significance, 4 variables (HR, BSA, RS_api/bas_, SD16-TTPRS) were substituted into the multivariate logistic regression model, which revealed that the recovered group was correlated with RS_api/bas_ (Odds Ratio: 0.380; 95% confidence interval: 0.155–0.934; P = 0.035) and SD16-TTPRS (Odds Ratio: 1.029; 95% confidence interval: 1.006–1.053; P = 0.012) (Table 3). The sensitivity and specificity of RS_api/bas_ and SD16-TTPRS for predicting recovered conditions were 76%, 67%, and 91%, 59%, with the area under the curve of 0.75 and 0.76, respectively. The cut-off value of RS_api/bas_ and SD16-TTPRS was 0.95 and 111 ms. The receiver operating characteristic curve analysis applied to identify the optimal cut-off point for predicting the recovered condition is shown in Fig. 6.

**Table 3:** Univariate and multivariate logistic regression

Variables	Univariate		
	OR	95% CI	P value
Heart rate	0.963	0.931–0.996	0.030
Body surface are	0.129	0.018–0.927	0.042
Hemoglobin	0.966	0.931–1.002	0.065
RS_api/bas_	0.321	0.140–0.736	0.007
SD16-TTPRS	1.030	1.010–1.051	0.004
CS_api/bas_	0.278	0.103–1.750	0.011
SD16-TTPCS	1.021	1.005–1.037	0.011

Variables	Multivariate		
	OR	95% CI	P value
Heart rate	0.989	0.952–1.027	0.555
Body surface area	0.455	0.044–4.709	0.509
Hemoglobin	–	–	–
RS_api/bas_	0.380	0.155–0.934	0.035
SD16-TTPRS	1.029	1.006–1.053	0.012

To find prognostic predictors for the recovered condition, univariate and multivariate logistic regression analysis were performed. Considering the collinearity and clinical significance, 4 variables (HR, BSA, RS_api/bas_, SD16-TTPRS) were included in the model, of which recovered group was correlated with RS_api/bas_ (Odds Ratio: 0.380; 95% confidence interval: 0.155–0.934; P = 0.035) and SD16-TTPRS (Odds Ratio: 1.029; 95% confidence interval: 1.006–1.053; P = 0.012)

### Recovered curve according to the predictive model

**Fig. 7:**
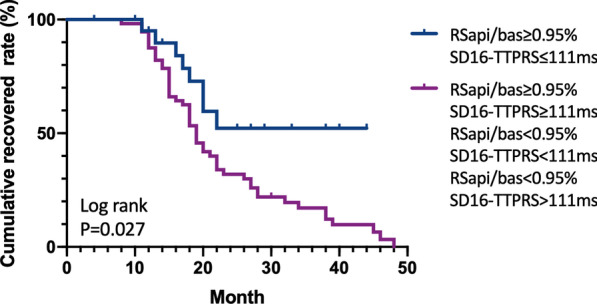
Kaplan Meier survival curve showed the highest recovered rate in RS_api/bas_ ≥ 0.95% and SD16-TTPRS ≤ 111 ms group (P = 0.027)

Further, Kaplan Meier survival analysis showed patients with RS_api/bas_ ≥ 0.95%, and SD16-TTPRS ≤ 111 ms had the highest recovery rate (65%, P = 0.027) (Fig. 7). However, the recovery rate in the remaining patients was only 11%.

### Intra- and inter-observer agreement

The results of the intra- and inter-observer analysis for CMR measurements are summarized in Table 4. The intra- and inter-observer agreement was excellent for LV volume, mass, strain and LGE parameters (all ICCs > 90%).

**Table 4:** Intra- and inter-observer variability for CMR measurements

Variables	Intra-observer (n = 20)		Inter-observer (n = 20)	
	ICC	95%CI	ICC	95 %CI
LVEDV, ml	0.980	0.949–0.992	0.983	0.956–0.993
LVESV, ml	0.997	0.993–0.999	0.996	0.990-0. 998
LVM, g	0.986	0.965–0.995	0.986	0.964–0.994
LV-GCS, %	0.988	0.970–0.995	0.985	0.963–0.994
LV-GRS, %	0.979	0.948–0.992	0.983	0.957–0.993
SD16-TTPRS, ms	0.936	0.839–0.975	0.922	0.802–0.969
LGE Quantitative, 2 SD% (n = 10)	0.920	0.714–0.980	0.915	0.696–0.989

The intra- and inter-observer agreement was excellent for LV volume, mass, strain and LGE parameters (all ICCs > 90%)

## Discussion

The purpose of this study was to evaluate if strain parameters assessed by CMR FT analysis could predict the reversible NDCM. Several new findings helped us to comprehensively understand the complicated prognosis of NDCM patients: (1) 27% NDCM patients had normalized LV size and systolic function after a median 36 months; (2) conventional LV systolic function parameters were similar between the recovered and unrecovered NDCM groups at baseline; (3) myocardial contractility at apical and intraventricular mechanical coordination were much better in recovered patients at baseline using CMR FT analysis, of which RS_api/bas_ and SD16-TTPRS were independent predictors of recovered condition.

Several studies have shown that the recovered condition incidence ranges from 14 to 41% in NDCM patients [3–5]. In our cohort, 27% of NDCM had a favorable outcome with normalization of LV size and systolic function after receiving standard treatments, which may shed light on the diversified prognosis of patients with NDCM.

LVEF, reflecting global LV systolic function, is a universal marker for routine risk stratification and therapeutic strategy decision in patients with NDCM. In this study, the CMR examination revealed no difference in LVEF at baseline in the two NDCM groups. LVEF only revealed the variation of global and accumulative LV volume and function, which later changes in the pathological process and lacks sensitivity and specificity to predict the disease’s subsequent prognosis. More delicate and accurate parameters are required to further discriminate NDCM patients.

LV deformation is expressed as strain, which represents the fractional or percentage change of a region of interest from its original dimension [19]. In this study, strain was applied to reflect the heart’s systolic contractility through relative displacement in three different dimensions (longitudinal, circumferential, and radial). It was found that global strains (GLS, GCS, and GRS) were similar between two NDCM groups, which at least implied no remarkable difference in global systolic contractility. Furthermore, it was also found that all the global and segmental strains dramatically declined in NDCM patients, while segmental CS and RS disproportionately dropped. A similar decline of basal and middle segments but relative apical preservation in recovered patients resulted in higher CS_api/bas_ and RS_api/bas_ compared to unrecovered. In other words, the heterogeneity of contractile injury exists in patients with NDCM. Similar to our results, Bach et al. [20] found the basilar-septum was a “sentinel” region injured earlier than other LV regions. In this study, the base-to-apex strain could help to differentiate the recovered NDCM from the unrecovered. With the spherical ventricular geometry variation, unrecovered patients had the most severely injured segments at the apical. The preserved apical strain is a compensatory mechanism that maintains LV systolic function. In the physiological state, the basal segment’s wall stress is higher than at the middle and apical segments due to the non-spherical ventricular geometry and the largest local radius of the LV curvature at the basal segment. For NDCM patients, the high wall stress at the basal segment will lead to cardiomyocyte necrosis and fibrosis. In addition, a greater diversity of myocardial fibers and matrix orientations at the apical segment compared with the basal segment could also contribute to the preserved deformation at the apical segment [21, 22]. This finding implied that the difference of base-to-apex strain among NDCM patients might indicate a different prognosis.

Moreover, the decreased contractility of the localized part and the loss of consistency of regional myocardium could also affect systolic function, which may directly weaken the cardiac pump. Therefore, more research is focusing on mechanical synchronism. Our study demonstrated that significant intraventricular mechanical dyssynchrony in regional longitudinal, circumferential, and radial dimensions were detected in unrecovered patients, and the difference was more obvious in the radial dimension. On the contrary, better mechanical synchronization was found in recovered patients; however, it was not as homogeneous as in control subjects, whose septum contracts slightly earlier than the lateral and inferolateral walls [22]. Intraventricular electrical conduction delay may exist in quite a few NDCM patients, while some HF patients with a narrow QRS duration (< 120 ms) may also exhibit significant mechanical dyssynchrony [23, 24]. Also, we found some patients with a long QRS duration (> 120 ms) but recovered LV size and systolic function. Our findings indicated that intraventricular mechanical dyssynchrony rather than a complete left bundle block might have a crucial role in the prognosis in NDCM patients [25]. The heart squeezes blood out of the LV cavity by any two of the opposing wall contracting almost at the same time. If any ventricular segment reaches peak strain earlier than others, there is an ineffective shifting of blood within the LV cavity resulting in smaller LV stroke volume. Moreover, the earlier contraction wall directly influences its opposing wall by overstretching the myocardium and causing increased wall stress and preload, which may further reduce its contractility [26]. Also, this may be one of the potential reasons why recovered patients with relatively better mechanical accordance are more likely to have the contractility recovered.

To the best of our knowledge, this is the first study that reported on the use of CMR FT strain analysis for prediction of functional recovery in NDCM patients. We found 27 % of NDCM patients had recovered LV size and systolic function in this cohort. RS_api/bas_ and SD16-TTPRS assessed by CMR at presentation may prove to be non-invasive parameters for prediction of recovery in patients with NDCM. Global and segmental strains of the recovered NDCM patients will be analyzed and reported in our post-study, which may offer greater insight into the disease.

The present study has several limitations. As this was a single-center study, the sample size was modest. Though RS_api/bas_ and SD16-FTPRS predict recovery, the area under the curve for each variable was below 0.80. More markers such as native T1 or extracellular volume fraction could not be performed, as both were not available in the majority of the patients. It remains unknown how much longer the recovered NDCM patients could maintain their morphologic and functional recovery. Long-term follow-up and molecular studies are needed to make a preliminary stratification of those patients at presentation.

## Conclusion

This study highlights the prevalence of the recovered condition in NDCM patients. Strain analysis using CMR FT imaging is considered a useful method to evaluate NDCM patients’ prognosis. Further prospective multicenter studies are needed to certify whether CMR strain assessed can be used to predict long-term prognosis in NDCM patients .

## Supplementary information

**Additional file 1.** Calculation of torsional shear angle from basal and apical slices.

## Data Availability

The datasets used and/or analyzed during the current study are available from the corresponding author on reasonable request.

## Abbreviations

ACEI: Angiotensin-converting enzyme inhibitors
ARB: Angiotensin receptor blocker
BSA: Body surface area
bSSFP: Balanced steady state free precision
CI: Cardiac index
CMR: Cardiovascular magnetic resonance
CS: Circumferential strain
CS_api/bas_: Ratio of apical and basal circumferential strain
ECG: Electrocardiogram
EDV: End-diastolic volume
eGFR: Estimated glomerular filtration rate
ESV: End-systolic volume
FT: Feature tracking
GCS: Global circumferential strain
GLS: Global longitudinal strain
GRS: Global radial strain
Hb: Hemoglobin
HF: Heart failure
HR: Heart rate
LA: Left atrium/left atrial
LGE: Late gadolinium enhancement
LS: Longitudinal strain
LV: Left ventricle/left ventricular
LVEDDI: Left ventricular end-diastolic diameter index
LVEDV: Left ventricular end-diastolic volume
LVEDVI: Left ventricular end-diastolic volume index
LVESV: Left ventricular end-systolic volume
LVESVI: Left ventricular end-systolic volume index
LVEF: Left ventricular ejection fraction
LVSVI: Left ventricular stroke volume
LVM: Left ventricular mass
LVMI: Left ventricular mass index
NDCM: Non-ischemic dilated cardiomyopathy
RS: Radial strain
RS_api/bas_: Ratio of apical to basal radial strain
RV: Right ventricle/right ventricular
RVEF: Right ventricular ejection fraction
SD16-TTPLS: Standard deviation of time to peak longitudinal strain
SD16-TTPCS: Standard deviation of time to peak circumferential strain
SD16-TTPRS: Standard deviation of time to peak radial strain
SVI: Stroke volume index
