# Supplementary material for: Strain parameters for predicting the prognosis of non‐ischemic dilated cardiomyopathy using cardiovascular magnetic resonance tissue feature tracking
Source: J Cardiovasc Magn Reson. 2021 Mar 15;23:21. doi: 10.1186/s12968-021-00726-3 (PMC7958458; doi:10.1186/s12968-021-00726-3)
Supplement: Supplementary file 1 — Additional file 1. Calculation of torsional shear angle from basal and apical slices. [file 12968_2021_726_MOESM1_ESM.docx]

**Additional Material.** Calculation of torsional shear angle from basal and apical slices. α_base_, α_apex_: rotation angle for A1→A2, B1→B2. β: torsional shear angle.
